# Enhanced Safety in Autonomous Driving: Integrating a Latent State Diffusion Model for End-to-End Navigation

**DOI:** 10.3390/s24175514

**Published:** 2024-08-26

**Authors:** De-Tian Chu, Lin-Yuan Bai, Jia-Nuo Huang, Zhen-Long Fang, Peng Zhang, Wei Kang, Hai-Feng Ling

**Affiliations:** 1 Field Engineering College, Army Engineering University of PLA, Nanjing 210007, China; detian_chu@aeu.edu.cn (D.-T.C.); linyuan_bai@aeu.edu.cn (L.-Y.B.); shuiyuantou2519@163.com (P.Z.); w_kang2023@163.com (W.K.); 2 School of Computing and Data Science, Xiamen University Malaysia, Sepang 43900, Malaysia; jasm4041@163.com; 3 School of Mathematics and Computer Sciences, Nanchang University, Nanchang 330031, China; zhenlongfang0808@163.com

**Keywords:** end-to-end driving, safe navigation, motion planning

## Abstract

Ensuring safety in autonomous driving is crucial for effective motion planning and navigation. However, most end-to-end planning methodologies lack sufficient safety measures. This study tackles this issue by formulating the control optimization problem in autonomous driving as Constrained Markov Decision Processes (CMDPs). We introduce an innovative, model-based approach for policy optimization, employing a conditional Value-at-Risk (VaR)-based soft actor-critic (SAC) to handle constraints in complex, high-dimensional state spaces. Our method features a worst-case actor to ensure strict compliance with safety requirements, even in unpredictable scenarios. The policy optimization leverages the augmented Lagrangian method and leverages latent diffusion models to forecast and simulate future trajectories. This dual strategy ensures safe navigation through environments and enhances policy performance by incorporating distribution modeling to address environmental uncertainties. Empirical evaluations conducted in both simulated and real environments demonstrate that our approach surpasses existing methods in terms of safety, efficiency, and decision-making capabilities.

## 1. Introduction

In the rapidly evolving field of autonomous driving, ensuring vehicle safety during the exploration phase is critical [1,2]. Traditional end-to-end methods often fail to guarantee safety in complex, high-dimensional environments [3,4]. The increased complexity of such scenarios makes sampling and learning inefficient and hinders the pursuit of globally optimal policies. Inadequate safety measures can lead to severe consequences, including system damage and significant threats to human life.

Reinforcement learning (RL) has achieved remarkable success across various domains [5,6]. Deep learning (DL) excels in perception, while RL is proficient in decision-making. The integration of DL and RL, known as deep reinforcement learning (DRL), addresses decision-making in complex obstacle avoidance scenarios. Unlike traditional motion planning methods, DRL can enhance adaptability and generalization across diverse scenarios, overcoming the limitations of conventional approaches and providing a more efficient and effective solution. Mnih et al. proposed a Deep Q-Network (DQN) model that combines convolutional neural networks and Q-learning from traditional RL to address high-dimensional perception-based decision problems [6]. This widely adopted approach serves as a primary driver for deep RL. DRL equips robots with perceptual and decision-making capabilities by processing input data to generate the output in an end-to-end manner [7]. The end-to-end motion planning approach treats the system holistically, enhancing its robustness [8,9]. Moreover, DRL can manage high-dimensional and nonlinear environments by utilizing neural networks to learn intricate state and action spaces [10,11]. In contrast, traditional heuristic algorithms require a manual design of the state and action spaces and may need rules to be redesigned for new scenarios, leading to algorithm limitations and performance bottlenecks. Previous research in RL and DL has shown progress in addressing complex scenarios, whereas traditional methods are hindered by their dependence on manual design for state and action spaces, resulting in adaptability issues and reduced performance in novel situations.

Recognizing these challenges, various methods have been proposed. One such method is the framework of Constrained Markov Decision Processes (CMDPs), which seeks to balance reward maximization and risk mitigation by optimizing the trade-off between exploration and exploitation [12,13]. Building on prior research, this study redefines the control optimization problem within the CMDP framework. We propose an innovative, model-based policy optimization framework that integrates the Augmented Lagrangian method, and latent diffusion models to effectively manage safety constraints in autonomous navigation tasks while optimizing navigation proficiency.

Our methodology begins with a latent variable model designed to produce extended-horizon trajectories, enhancing our system’s ability to predict future states accurately. To further strengthen the safety, we incorporate a conditional Value-at-Risk (VaR) within the soft actor-critic (SAC) framework, ensuring safety constraints are met through the Augmented Lagrangian method for efficiently solving the safety-constrained optimization challenges.

For extreme risk scenarios, our approach includes a worst-case scenario planning method that employs a “worst-case actor” during policy exploration to ensure the safest outcomes in potentially dangerous conditions. To enhance performance, we integrate a latent diffusion model for state representation learning, refining our system’s ability to interpret complex environmental data.

The efficacy of our proposed approach is validated through extensive experiments. Our empirical findings verify our approach’s capability to manage and mitigate risks effectively in high-dimensional state spaces, enhancing the safety and reliability of autonomous vehicles in complex driving environments.

Our main contributions are as follows.

- We integrate latent diffusion models for state representation learning, enabling the forecasting of future observations, rewards, and actions. This capability allows for the simulation of future trajectories within the model framework, facilitating the proactive assessment of rewards and risks through controlled model roll-outs.
- We further extend our approach to include advanced prediction of future state value distributions, incorporating the estimation of worst-case scenarios. This ensures that our model predicts and prepares for potential adverse conditions, enhancing system reliability.
- Our experimental results demonstrate the efficacy of our proposed approaches in simulated and real-world environments, which can also guarantee safe policy exploration in unpredictable scenarios.

## 2. Related Work

### 2.1. Safe Reinforcement Learning

Safe reinforcement learning (Safe RL) integrates safety measures with the standard learning process for agents in complex environments [14]. Developing safe and reliable autonomous driving systems necessitates a comprehensive focus on safety throughout control and decision-making processes. Safe RL is a powerful tool for training controllers and planners to navigate dynamic environments while adhering to safety constraints.

Safe RL algorithms tackle the safety challenge through various methods. Examples include Constrained Policy Optimization (CPO) [8], which maximizes policies within clearly defined safety constraints to avoid unsafe actions. Trust Region Policy Optimization (TRPO) [15] and Proximal Policy Optimization (PPO) [16] refine these algorithms to integrate safety considerations by implementing penalty terms or barrier functions. Gaussian Processes (GPs) model [17] environmental uncertainty, enabling safe exploration by quantifying the risk associated with different actions. Model Predictive Control (MPC)-based safe RL [18,19] predicts future states and maximizes actions over a finite horizon while complying with safety constraints.

Safe RL plays a critical role in ensuring the safety of autonomous driving. Its applications include high-level decision-making tasks such as lane changing [13] and route planning [20], factoring in the behavior of surrounding vehicles and potential conflicts. Low-level control merges safe RL with conventional methods like MPC, ensuring adherence to safety constraints while following the planned path [21,22]. Combining RL with rule-based systems that encode traffic laws ensures that learned policies comply with rules. Safety verification techniques provide assurances that learned policies maintain safety in the presence of uncertainties and dynamic obstacles [23,24].

The variety of safe RL algorithms and their applications highlight their crucial role in developing adaptable and secure control systems. Ongoing advancements in these approaches will be essential for the successful real-world deployment of autonomous vehicles.

### 2.2. Reinforcement Learning with Latent State

Latent dynamic models, pivotal in modeling time-series data within reinforcement learning, enable a deep understanding of concealed states and dynamic changes in intricate environments [7,25,26]. These models capture crucial relationships between unobservable internal states and observed data, substantially enhancing the predictive capabilities of RL agents.

In the context of RL, latent dynamic models operate under probabilistic frameworks, utilizing Bayesian inference or maximum likelihood estimation to accurately deduce both model parameters and hidden states [27,28]. These frameworks facilitate environment modeling that aligns with the probabilistic nature of real-world dynamics.

Mathematically, latent dynamic models are characterized by the following:

- **State Transition Equation:**
  $$s_{t+1}=f\left(s_{t},a_{t},\epsilon_{t}\right)$$
  where $s_{t}$ denotes the latent state at time *t*; $a_{t}$ signifies the action taken; $\epsilon_{t}$ represents the stochasticity inherent in the environment. The function *f* may be deterministic or stochastic, encapsulating the uncertainty of state transitions.
- **Observation Equation:**
  $$o_{t}=g\left(s_{t},\delta_{t}\right)$$
  where $o_{t}$ represents the observed output linked to the hidden state $s_{t}$; $\delta_{t}$ signifies the observation noise, linking theoretical models to real-world observations.
- **Reward Function:**
  $$r_{t}=R\left(s_{t},a_{t}\right)$$
  defines the immediate reward received after executing action $a_{t}$ in state $s_{t}$, essential for policy optimization in RL.

The primary objective of using latent dynamic models in RL is to infer the sequence of hidden states $s_{1},s_{2},\ldots ,s_{T}$ from observations $o_{1},o_{2},\ldots ,o_{T}$, actions $a_{1},a_{2},\ldots ,a_{T}$, and rewards $r_{1},r_{2},\ldots ,r_{T}$. This modeling approach predicts future states or actions and integrates environmental dynamics to enhance decision-making processes and optimize policy outcomes [11,29,30]. Additionally, these models provide a deeper understanding of environmental complexities, enabling RL agents to make more informed and effective decisions [31,32].

### 2.3. Diffusion-Model-Based Reinforcement Learning

Recent advances in diffusion models have significantly impacted RL, especially offline RL, where interaction with the environment is limited. Originally successful in generative tasks, diffusion models are adapted to address challenges such as distributional shift and extrapolation errors in offline settings [33,34,35].

Research [36] indicates that diffusion probabilistic models can effectively generate plausible future states and actions, facilitating robust policy learning from static datasets. Additionally, latent diffusion models (LDMs) reduce computational demands and enhance learning efficiency by encoding trajectories into a compact latent space before diffusion, thus capturing complex decision dynamics [37]. These approaches improve the stability and efficacy of Q-learning algorithms by ensuring that generated actions remain within the behavioral policy’s support, mitigating the risk of policy deviation due to poor sampling [38].

Integrating diffusion techniques into RL frameworks represents a promising frontier for developing more capable and reliable autonomous systems, particularly in environments where conventional learning approaches are inadequate; however, how to improve safety is the main key problem.

## 3. Problem Modeling

Autonomous navigation involves modeling the interaction between an autonomous agent and a dynamic, uncertain environment using a finite-horizon Markov Decision Process (MDP), denoted by the tuple M∼(S,O,A,P,r,γ). Here, $S\subset R^{n}$ represents a high-dimensional continuous state space, and $A\subset R^{m}$ represents the action space. State transitions, defined by $s_{t+1}∼P\left(\cdot |s_{t},a_{t}\right)$, capture the environment’s stochastic characteristics.

Observations O, derived from the state space, are high-dimensional images captured by sensors and analyzed via a latent diffusion model to refine state representation. This detailed comprehension of environmental dynamics is vital for navigating complex scenarios. The reward function r:S×A×S→R and the discount factor γ∈[0,1] inform the agent’s policy $\pi_{\theta }$, which produces actions based on analyzed observations $o_{t}$.

We prioritize safety by incorporating a conditional VaR metric into our decision-making framework, addressing worst-case scenarios through latent state diffusion for robust state representation learning. Safety protocols are formalized with a subset $S_{u}\subset R^{n}$, where entering a state $s_{t}\in S_{u}$ signifies a potential safety breach, monitored by a safety function κ. The objective extends beyond maximizing cumulative rewards to minimizing safety violations.

(1) $$\max_{\theta }J\left(\pi_{\theta }\right)=E_{P\left(\cdot |s_{t},a_{t}\right)}\left[\sum_{t=0}^{T}\gamma^{t}r\left(s_{t},a_{t},s_{t+1}\right)\right],\;s.t.\;,\sum_{t=0}^{T}\kappa \left(s_{t}\right)\leq D,a_{t}∼\pi_{\theta }\left(\cdot |o_{t}\right)$$

where $\kappa \left(s_{t}\right)\in \left\{0,1\right\}$ indicates safety violations, with D∈R representing the maximum allowable safety violations, aiming for D→0 to enhance operational safety. This safety-constrained MDP framework enables the agent to learn navigation policies that maximize efficiency while ensuring safety, effectively balancing high performance with adherence to critical safety constraints.

## 4. Methodology

### 4.1. Constrained Markov Decision Process Formulation

Reinforcement learning involves continuous interaction between the agent and the environment. This study focuses on the safety of autonomous driving, requiring a trade-off between reward and safety. We formulate the control optimization problem as a Constraint Markov Decision Process (CMDP), defined by $\left(S,A,p,r,c,d,\gamma \right)$, including the state space, action space, transition model, reward, cost, constraint, and discount factor. At each step, the agent receives a reward *r* and cost *c*. The optimization objective is to maximize the reward subject to the safety constraint in Equation (2).

(2) $$\begin{array}{cc} \; & \max_{\pi }\underset{\left(s_{t},a_{t}\right)∼\tau_{\pi }}{E}\left[\sum_{t}\gamma^{t}r\left(s_{t},a_{t}\right)\right]\\ \; & s.t.\underset{\left(s_{t},a_{t}\right)∼\tau_{\pi }}{E}\left[\sum_{t}\gamma^{t}c\left(s_{t},a_{t}\right)\right]\leq d \end{array}$$

where π and $\tau_{\pi }$ denote the policy and the trajectory distribution of the policy, respectively. Current CMDP methods, while effective, often struggle with the high-dimensional, nonlinear state and action spaces inherent in complex traffic environments. Moreover, it lacks a more generalizable safety constraint. Therefore, an improved method is introduced in the following sections.

### 4.2. Build the Latent Diffusion Model for State Representation

In this section, we elaborate on the latent state space representation and the diffusion process employed in our Enhanced Safe Navigation Autonomous Driving (ESAD-LEND) model.

**Figure 1:**
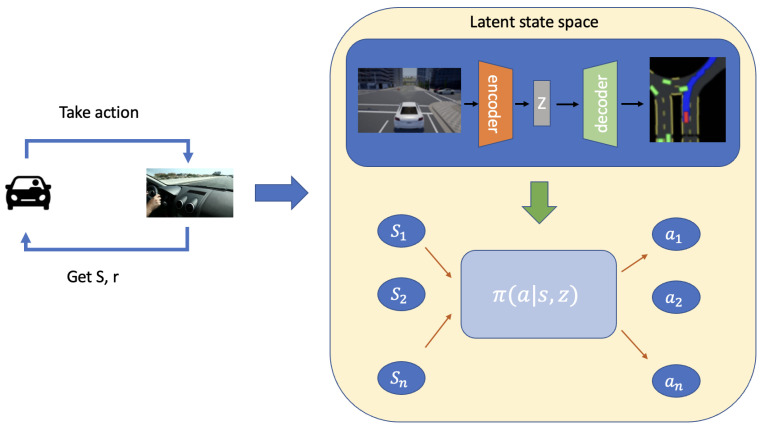
Our proposed framework, the Enhanced Safe Navigation Autonomous Driving model with Latent State End-to-Navigation Diffusion model (ESAD-LEND).

As shown in Figure 1, the model begins by capturing the state (*S*) and reward (*r*) from the environment based on the actions taken by the autonomous driving agent. This information is then encoded into a latent state (*z*) using a Variational Autoencoder (VAE). The VAE consists of an encoder and a decoder, where the encoder transforms the input state into a latent representation, and the decoder attempts to reconstruct the original input from this latent space. The latent state space is depicted in the top right section of Figure 1, where the encoder encodes the observed state into the latent representation *z*. This representation *z* is then combined with the current state (*S*) to inform the policy network π(a|S,z), which generates the appropriate action (*a*) to be taken by the autonomous vehicle. The purpose of using a VAE is to ensure that the latent space captures the essential features of the input state while allowing for effective reconstruction, thereby creating a robust and informative latent representation.

**Figure 2:**
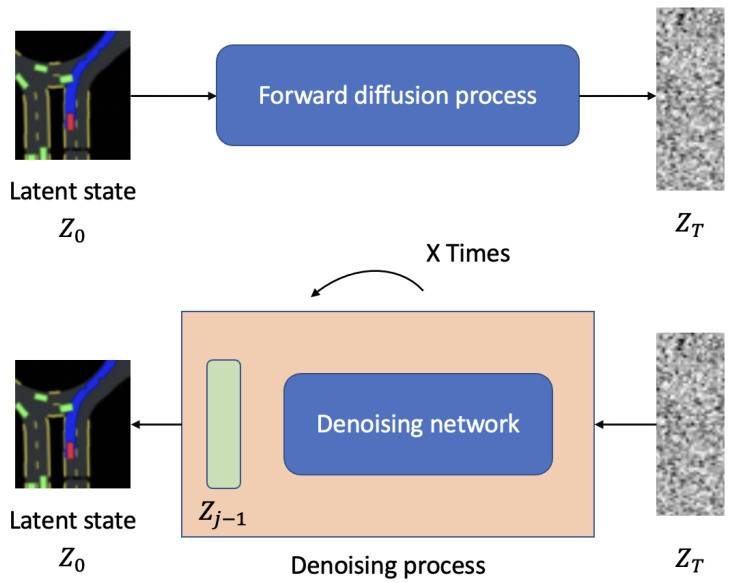
The training process for a diffusion-based model.

Furthermore, as shown in Figure 2, the training process for the diffusion-based model is illustrated. The latent state $z_{0}$, derived from the VAE encoder in Figure 1, undergoes a forward diffusion process. This process gradually adds noise to the latent state, resulting in a highly noisy latent state $z_{T}$. The noisy latent state $z_{T}$ is then passed through a denoising network, which iteratively denoises it over multiple steps to recover the original latent state $z_{0}$. This iterative denoising process ensures that the latent space is robust to noise and can effectively capture the essential features required for safe navigation. The forward diffusion and subsequent denoising make the latent representation resilient to variations and disturbances, enhancing the model’s performance in real-world scenarios.

The new method proposed in this paper integrates the latent model, as illustrated in Figure 2, containing three critical components: a representation model, a transition model, and a reward model. We consider the latent state as extracted and depicted in Figure 1. These models are trained to work in synergy for the accurate prediction and navigation in complex environments, utilizing both observed data and imagined trajectories.

- **Representation Model:** The representation model establishes a robust latent space based on past experiences. The representation model is formalized as $p(s_{\tau }|s_{\tau -1},a_{\tau -1},o_{\tau })$, predicting the next state by integrating information from the current state, action, and observation. The representation loss is quantified by assessing the accuracy of state and reward predictions.
- **Transition Model:** This model outputs a Gaussian distribution, defined as $q(s_{\tau }|s_{\tau -1},a_{\tau -1})$. The transition model’s accuracy is evaluated using the Kullback–Leibler (KL) divergence between the predicted and actual distributions, signifying the latent imagination and the environment’s real response, respectively.
- **Reward Model:** The reward model enhances learning by computing expected rewards based on the current state, $q(r_{\tau }|s_{\tau })$. This model is crucial for the agent to enhance actions and maximize environmental returns.

In our framework, *p* denotes the distribution from environment interactions, while *q* represents latent space predictions. Time steps in the latent space are indexed by τ. Our model’s core innovation lies in its ability to create and refine a latent imagination space for predicting future trajectories. This enables the agent to explore safely and learn optimal behaviors by iteratively refining the latent space to avoid unsafe states and maximize efficiency.

### 4.3. Build Safety Guarantee

**Figure 3:**
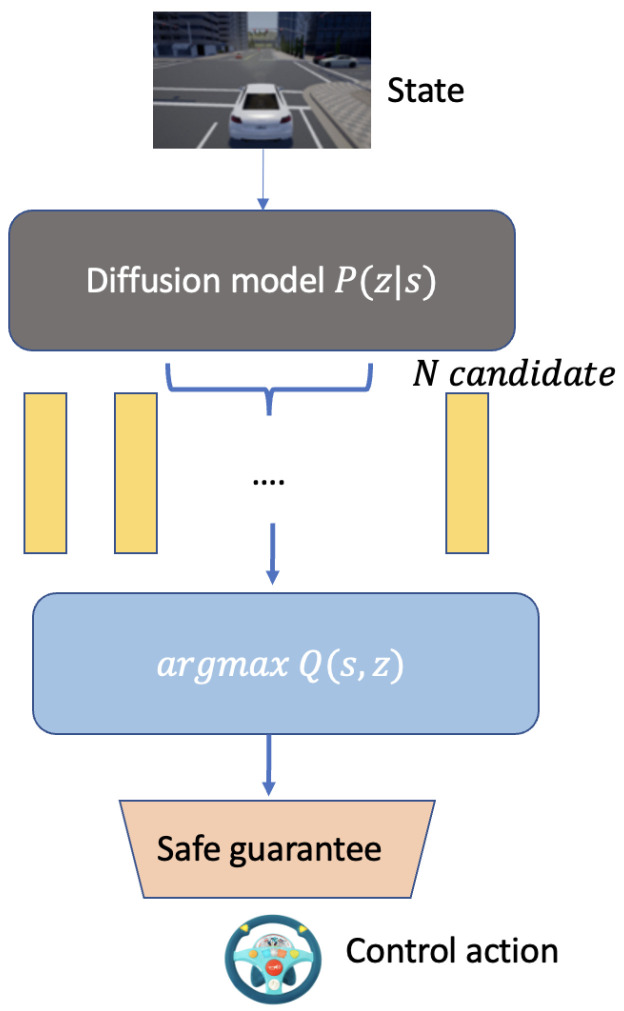
The policy generation process in a diffusion-based control system.

Figure 3 illustrates the policy generation process within a diffusion-based control system. The process begins with the state of the environment, input into a diffusion model denoted as P(z|s). This model generates multiple candidate latent states, representing potential actions or decision paths.

These candidates are evaluated using a Q-function, argmaxQ(s,z), which chooses the optimal latent state for action execution by maximizing expected rewards and ensuring compliance with safety standards. The chosen action is optimal in performance and satisfies predefined safety criteria, ensuring adherence to safety standards before execution.

This structured approach guarantees robust safety by integrating performance optimization and rigorous safety compliance, which is crucial for autonomous systems operating in dynamic and uncertain environments.

A similar approach can be found in latent imagination, such as Dreamer [39], which achieved excellent performance. However, Dreamer did not consider safety constraints. Although Dreamer reached high reward targets, the absence of safety measures could lead to irreversible incidents in pursuit of higher rewards. Therefore, it is crucial to introduce safety constraints to balance reward and risk.

In our latent imagination framework, we leverage distributional reinforcement learning to address the safety-constrained RL problem [40]. Instead of following a policy π to obtain an action value, distributional RL focuses on the expectation of value and cost [41]. We concentrate on the actor-critic policy and develop a model-based algorithm incorporating latent imagination. The soft actor-critic (SAC) [42] introduces maximum entropy to balance exploitation and exploration.

(3) $$\pi^{*}=\underset{\pi }{\mathrm{argmax}}\sum_{t=0}^{T}E_{\left(s_{t},a_{t}\right)∼\tau_{\pi }}\left[\gamma^{t}\left(r\left(s_{t},a_{t}\right)+\beta H\left(\pi \left(.|s_{t}\right)\right)\right]\right.$$

where $\pi^{*}$ denotes the optimal policy, and β represents the stochasticity of π.

Agents can learn the optimal policy without safety concerns; however, irreversible situations such as collisions are unacceptable in autonomous driving. We address these safety constraints by formulating them using a Lagrangian method with constraints.

(4) $$\begin{array}{cc} \; & \max_{\pi }\underset{\left(s_{t},a_{t}\right)∼\rho_{\pi }}{E}\left[\sum_{t}\gamma^{t}r\left(s_{t},a_{t}\right)\right]\\ s.t. & \left\{\begin{array}{c} \underset{\left(s_{t},a_{t}\right)∼\rho_{\pi }}{E}\left[\sum_{t}\gamma^{t}c\left(s_{t},a_{t}\right)\right]\leq d\\ \underset{\left(s_{t},a_{t}\right)∼\rho_{\pi }}{E}\left[-\log\left(\pi_{t}\left(a_{t}|s_{t}\right)\right)\right]\geq H_{0}\;\forall t\\ h(s_{t})\leq 0\;(\mathrm{Safety}\;\mathrm{Constraint})\\ h\left(s_{t+1}\right)\leq \left(1-\alpha \right)h\left(s_{t}\right)\;(\mathrm{Control}\;\mathrm{Barrier}\;\mathrm{Function}) \end{array}\right. \end{array}$$

To enhance the utilization of safety constraints, a barrier function is employed in distributional RL to adjust the risk assessment, allowing the agent to determine optimal exploration and conservatism strategies. In this formulation, for a constraint with relative degree *m*, the generalized control barrier function is defined as follows:

(5) $$h\left(s_{t+m}\right)\leq \left(1-\alpha \right)h\left(s_{t}\right)$$

For a specified risk level α, we optimize the policy until $\Gamma_{\pi }$ meets the following condition:

(6) $$\begin{array}{c} \Gamma_{\pi }\left(s,a,\alpha \right)≐CVaR_{\alpha }=Q_{\pi }^{c}\left(s,a\right)+\alpha^{-1}\phi \left(\Phi^{-1}\left(\alpha \right)\right)\sqrt{V_{\pi }^{c}\left(s,a\right)}\\ \Gamma_{\pi }\left(s_{t+m},a_{t+m},\alpha \right)\leq \left(1-\alpha \right)\Gamma_{\pi }\left(s_{t},a_{t},\alpha \right) \end{array}$$

where α signifies the conservativeness coefficient.

Next, the standard soft actor-critic (SAC) framework is enhanced by integrating safety constraints using a barrier function, which adjusts risk assessment and balances exploration with conservatism.

### 4.4. VaR-Based Soft Actor-Critic for Safe Exploration

**Figure 4:**
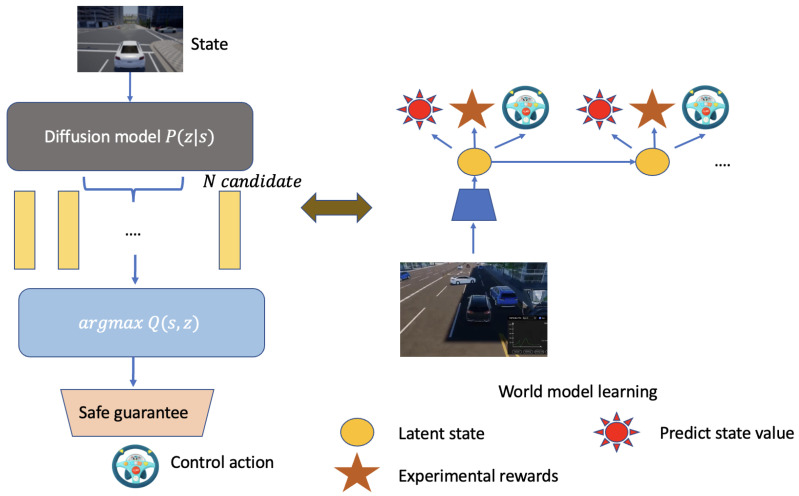
The world model learning process in our autonomous system.

As shown in Figure 4, it illustrates the process of world model learning within our autonomous system, which is pivotal for the development and refinement of our latent imagination model. Following Figure 3, the *world model learning process* involves iterative updates to the representation, transition, and reward models. The transition model, in particular, is used to predict future states and rewards, which are essential for planning and decision-making.

As depicted in Algorithm 1, we construct the latent imagination through iterative updates of the representation, transition, and reward models. Future trajectories are predicted using the transition model, serving as the basis for the latent imagination in continuous actor-critic policy optimization. Upon defining a specific risk-awareness level, new safety constraints are introduced. By considering the expected cumulative cost and reward, the model is updated using distributional RL. Following iterative learning within the latent imagination over discrete time steps, the agent interacts with the environment to acquire new reward and cost data.
**Algorithm 1** Pseudocode for ESAD-LEND1:Inputs: Initial parameters α,ψ,μ,η,θ, and τ2:Initialize: Target networks: $\langle \bar{\psi },\bar{\mu },\bar{\eta }\rangle \leftarrow \langle \psi ,\mu ,eta\rangle$3:Initialize: Dataset D with *S* random seed episodes.4:**while** not onverged **do**5:     **for** update step t=1…T  **do**6:         // World Model learning with Latent Diffusion Representation7:         Sample a sequence batch of ${\left\{\left(o_{t},a_{t},r_{t},c_{t}\right)\right\}}_{t=k}^{k+L}$ from D.8:         Encode observed states to latent space: $z_{t}∼q_{\theta }\left(z_{t}|o_{t},h_{t-1}\right)$ using a latent diffusion model.9:         Predict next state and reward using latent representations: $h_{t}=f_{\theta }\left(h_{t-1},z_{t},a_{t-1}\right)$.10:        Optimize θ by minimizing the equation L(θ).11:        // Behavior learning12:        Imagine trajectories ${\left\{\left(s_{\tau },a_{\tau }\right)\right\}}_{\tau =t}^{t+H}$ from each $s_{t}$.13:        Compute safety measure $\Gamma_{\pi }\left(s,a,\alpha \right)$ based on $\Gamma_{\pi }\left(s,a,\alpha \right)≐CVaR_{\alpha }=Q_{\pi }^{c}\left(s,a\right)+\alpha^{-1}\phi \left(\Phi^{-1}\left(\alpha \right)\right)\sqrt{V_{\pi }^{c}\left(s,a\right)}$14:        $\psi_{i}\leftarrow \psi_{i}-\lambda_{R}{\hat{\nabla }}_{\psi_{i}}J_{R}\left(\psi_{i}\right)\;\mathrm{for}\;i\in \left\{1,2\right\}$15:        $\theta \leftarrow \theta -\lambda_{\pi }{\hat{\nabla }}_{\theta }J_{\pi }\left(\theta \right)$16:        $\mu ,\eta ,\beta ,\kappa \leftarrow \mu -\lambda_{C}{\hat{\nabla }}_{\mu }J_{C}\left(\mu \right),\eta -\lambda_{V}{\hat{\nabla }}_{\eta }J_{V}\left(\eta \right),\beta -\lambda_{\beta }{\hat{\nabla }}_{\beta }J_{e}\left(\beta \right),\kappa -\lambda_{\kappa }{\hat{\nabla }}_{\kappa }J_{s}\left(\kappa \right)$17:        // Update target network weights18:        ${\bar{\psi }}_{i}\leftarrow \tau \psi_{i}+\left(1-\tau \right){\bar{\psi }}_{i}\;\mathrm{for}\;i\in \left\{1,2\right\}$19:        $\bar{\mu },\bar{\eta }\leftarrow \tau \mu +\left(1-\tau \right)\bar{\mu },\tau \eta +\left(1-\tau \right)\bar{\eta }$20:     **end for**21:    // Environment interaction22:    $o_{1}\leftarrow$ env · reset ()23:     **for** time step t=1…T  **do**24:        Update state $s_{t}$ using latent diffusion model: $s_{t}∼p_{\theta }\left(s_{t}|s_{t-1},a_{t-1},z_{t}\right)$.25:        Compute action $a_{t}∼\pi_{\theta }\left(a_{t}|s_{t}\right)$ using the action model.26:        Add exploration noise to action.27:        $r_{t},o_{t+1}\leftarrow \mathrm{env}\cdot \mathrm{step}\left(a_{t}\right)$.28:     **end for**29:    Add experience to dataset $D\leftarrow D\cup \left\{{\left(o_{t},a_{t},r_{t}\right)}_{t=1}^{T}\right\}$.30:**end while**

## 5. Experiments

### 5.1. Environmental Setup

#### Experimental Setup in CARLA Simulator

In our study, we employed the CARLA simulator to construct and evaluate various safety-critical scenarios challenging the response capabilities of autonomous driving systems. CARLA offers a comprehensive, open-source environment specifically designed for autonomous driving research, featuring realistic urban simulations.

**Specific scenario**

**Figure 5:**
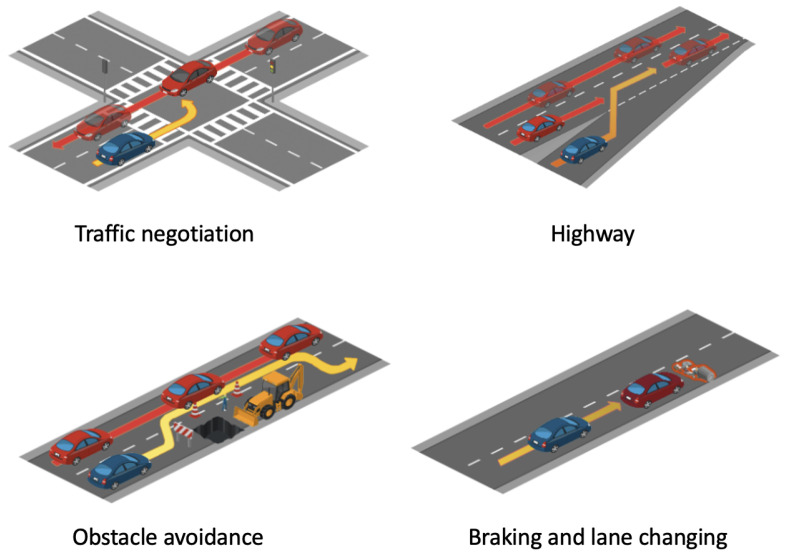
Illustration of various safety-critical scenarios developed to assess the response capabilities of autonomous driving systems.

We designed specific scenarios in CARLA to evaluate various aspects of autonomous vehicle behavior, as illustrated in Figure 5. These scenarios included the following:

- **Traffic Negotiation:** Multiple vehicles interact at a complex intersection, testing the vehicle’s ability to negotiate right-of-way and avoid collisions.
- **Highway:** Simulates high-speed driving conditions with lane changes and merges, assessing the vehicle’s decision-making speed and accuracy.
- **Obstacle Avoidance:** Challenges the vehicle to detect and navigate around sudden obstacles such as roadblocks.
- **Braking and Lane Changing:** Tests the vehicle’s response to emergency braking scenarios and rapid lane changes to evade potential hazards.

These scenarios are essential for validating the robustness and reliability of safety protocols in autonomous vehicles across diverse urban conditions.

**Urban Driving Environments**

**Figure 6:**
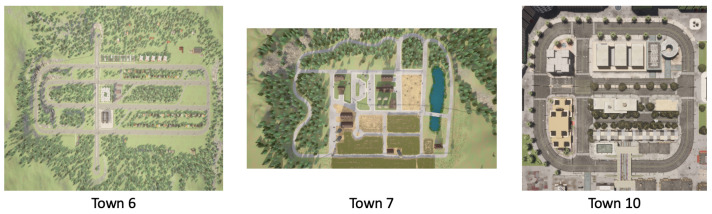
Aerial views of urban environments in the CARLA simulator: Town 6, Town 7, and Town 10. Town 6 features a typical urban grid with straightforward navigation challenges; Town 7 includes winding roads and a central water feature, introducing complexity to navigation tasks; and Town 10 offers a dense urban environment with numerous intersections and limited maneuvering space.

Additionally, we tested the vehicles across three distinct urban layouts in CARLA, as depicted in Figure 6. These towns were selected based on the following criteria:

- **Town 6:** Features a typical urban grid that simplifies navigation while testing adherence to basic traffic rules.
- **Town 7:** Incorporates winding roads and a central water feature, introducing complexity to navigation tasks and necessitating advanced path planning.
- **Town 10:** Represents a dense urban environment with numerous intersections and limited maneuvering space, ideal for testing advanced navigation strategies.

The comprehensive simulation environments offered by CARLA, coupled with the designed scenarios, facilitate thorough testing of autonomous driving algorithms, ensuring their safe and efficient operation in real-world conditions.

Considering the generalizability of our model, we selected Town 10 for its urban complexity and Town 7 for its natural landscape as the primary training environment. In random scenarios, vehicles, including the ego vehicle, can appear at any location on the map. In fixed scenarios, vehicle appearances are limited to a predefined range. However, both scenarios adhere to CARLA’s randomization protocols in each training and evaluation episode.

**Real-world scenario construction**

**Figure 7:**
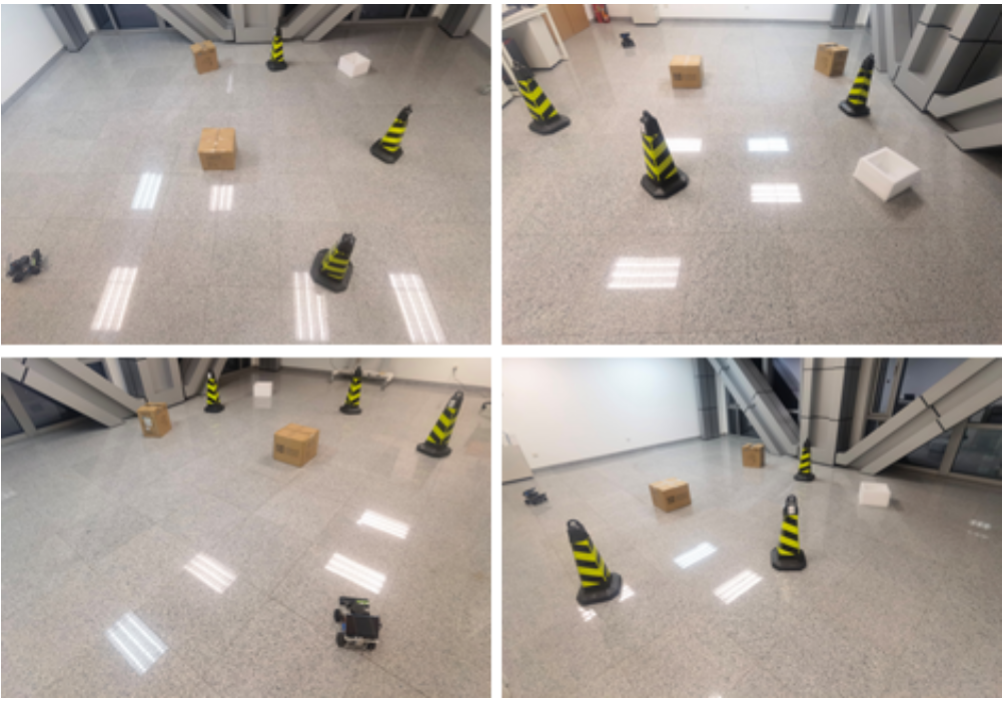
Real-world setups testing obstacle avoidance in autonomous systems.

As illustrated in Figure 7, these experiments evaluated the autonomous system’s ability to detect, negotiate, and navigate around obstacles. The setups ranged from simple configurations with minimal obstacles to intricate scenarios featuring densely packed obstacles, assessing the system’s adaptability to dynamically changing and physically constrained environments. The robot is named “Ackerman/Differential ROS car robot” and is controlled by a Jetson Nano running ROS. It includes a depth camera for visual perception, but not for speech interaction. The laser rangefinder (Lidar M10P, Riegl) has a measurement radius of 30 m, a scanning frequency of 12 Hz, and a sampling frequency of 20,000 Hz. The output contains angle and distance information. The robot is driven by brushless motors and uses a 360° scanning range to measure distance. Despite transmission delays, our experiments revealed that these delays had minimal impact on the overall performance metrics, including the collision rate and navigation efficiency.

The main objective of these real-world tests was to validate the robustness and reliability of the navigation algorithms and their associated safety mechanisms. Through these tests, our goal was to ensure that the autonomous systems could effectively detect and avoid immediate physical obstacles while maintaining adherence to safety standards across diverse environmental conditions.

### 5.2. Design of the Reward Function

Our autonomous system utilized a complex reward function aimed at optimizing navigation efficiency and safety. The reward function is segmented into multiple components that collectively ensure the vehicle adheres to operational standards:

#### 5.2.1. Velocity Compliance Reward ($R_{v}$)

This reward is awarded for maintaining a specified target velocity, encouraging efficient transit and fuel economy:

(7) $$R_{v}=\left\{\begin{array}{cc} 1 & \mathrm{if}\;v_{\mathrm{current}}=v_{\mathrm{target}}\\ \frac{1}{1+\lambda \left|v_{\mathrm{current}}-v_{\mathrm{target}}\right|} & \mathrm{otherwise} \end{array}\right.$$

where $v_{\mathrm{current}}$ represents the vehicle’s current velocity, $v_{\mathrm{target}}$ denotes the target velocity, and λ is a parameter that penalizes deviations from this target.

#### 5.2.2. Lane Maintenance Reward ($R_{l}$)

This reward incentivizes the vehicle to stay within the designated driving lane:

(8) $$R_{l}=\left\{\begin{array}{cc} 1 & \mathrm{if}\;d_{\mathrm{offset}}=0\\ -1 & \mathrm{if}\;d_{\mathrm{offset}}>d_{\max}\\ \frac{d_{\max}-d_{\mathrm{offset}}}{d_{\max}} & \mathrm{otherwise} \end{array}\right.$$

where $d_{\mathrm{offset}}$ denotes the lateral displacement from the lane center, and $d_{\max}$ represents the threshold beyond which penalties are applied.

#### 5.2.3. Orientation Alignment Reward ($R_{o}$)

This component imposes penalties on the vehicle for incorrect heading angles:

(9) $$R_{o}=\frac{1}{1+\mu \left|\theta_{\mathrm{current}}-\theta_{\mathrm{ideal}}\right|}$$

where $\theta_{\mathrm{current}}$ represents the vehicle’s current orientation, $\theta_{\mathrm{ideal}}$ denotes the ideal orientation along the road, and μ is a constant that determines the strictness of the alignment requirement.

#### 5.2.4. Exploration Incentive Reward ($R_{e}$)

An innovative component introduced to promote the exploration of less-traveled paths, thereby enhancing the robustness of the navigation strategy:

(10) $$R_{e}=\exp\left(-\nu \cdot n_{\mathrm{visits}}\right)$$

where $n_{\mathrm{visits}}$ denotes the count of times a specific path or region has been traversed, and ν is a decay factor that diminishes the reward with repeated visits.

#### 5.2.5. Composite Reward Calculation

The overall reward ($R_{\mathrm{total}}$) is a composite measure, defined as follows:

(11) $$R_{\mathrm{total}}=\omega_{v}\cdot R_{v}+\omega_{l}\cdot R_{l}+\omega_{o}\cdot R_{o}+\omega_{e}\cdot R_{e}$$

where $\omega_{v}$, $\omega_{l}$, $\omega_{o}$, and $\omega_{e}$ are weights that prioritize different aspects of the reward structure according to strategic objectives.

### 5.3. Evaluation Metrics

Inspired by [43], to rigorously assess the performance of autonomous driving systems in our simulation, a comprehensive set of metrics is utilized, which encompasses the various facets of driving quality, including safety, efficiency, and adherence to rules. The metrics are defined as follows:

1. **Route Completion (RC):** This metric quantifies the percentage of each route completed by the agent without intervention. It is defined as follows:
  (12) $$RC=\frac{1}{N}\sum_{i=1}^{N}R_{i}\times 100\%$$
  where $R_{i}$ represents the completion rate for the *i*-th route. A penalty applies if the agent deviates from the designated route, reducing RC proportionally to the off-route distance.
2. **Infraction Score (IS):** Capturing the cumulative effect of driving infractions, this score uses a geometric series, with each infraction type assigned a specific penalty coefficient:
  (13) $$IS=\prod_{j=\{\mathrm{Ped},\;\mathrm{Veh},\;\mathrm{Stat},\;\mathrm{Red}\}}{\left(p_{j}\right)}^{{\#\mathrm{infractions}}_{j}}$$
  Coefficients are set as $p_{\mathrm{Ped}}=0.50$, $p_{\mathrm{Veh}}=0.60$, $p_{\mathrm{Stat}}=0.65$, and $p_{\mathrm{Red}}=0.70$ for infractions involving pedestrians, vehicles, static objects, and red lights, respectively.
3. **Driving Score (DS):** This primary evaluation metric combines route completion with infraction penalties:
  (14) $$DS=\frac{1}{N}\sum_{i=1}^{N}R_{i}\times P_{i}$$
  where $P_{i}$ is the penalty multiplier for infractions on the *i*-th route.
4. **Collision Occurrences (COs):** This metric quantifies the frequency of collisions during autonomous driving, providing a key measure of the safety and reliability of the driving algorithm. A lower CO value indicates better collision avoidance, which is critical for the safe operation of autonomous vehicles. This metric is defined as follows:
  (15) $$CO=\frac{\mathrm{Number}\;\mathrm{of}\;\mathrm{Collisions}}{\mathrm{Total}\;\mathrm{Distance}\;\mathrm{Driven}}\times 100$$
  where the Number of Collisions denotes the total count of collisions encountered by the autonomous vehicle, and the Total Distance Driven signifies the total distance covered by the vehicle during testing. This metric is expressed as a percentage to standardize the measure across various distances driven.
5. **Infractions per Kilometer (IPK):** This metric normalizes the number of infractions by the distance driven, providing a measure of infractions per unit distance:
  (16) $$IPK=\frac{\sum_{i=1}^{N}I_{i}}{\sum_{i=1}^{N}K_{i}}$$
  where $I_{i}$ denotes the number of infractions on the *i*-th route, and $K_{i}$ represents the distance driven on the *i*-th route.
6. **Time to Collision (TTC):** This metric estimates the time remaining before a collision occurs, assuming the current velocity and trajectory of the vehicle and any object or vehicle in its path remain unchanged. It critically measures the vehicle’s ability to detect and react to potential hazards in its immediate environment:
  (17) $$TTC=\min\left(\frac{d}{v_{\mathrm{rel}}}\right)$$
  where *d* represents the distance to the nearest object in the vehicle’s path, and $v_{\mathrm{rel}}$ is the relative velocity towards the object. A lower TTC value signifies a higher immediate risk, necessitating more urgent responses from the system.
7. **Collision Rate (CR):** This metric quantifies the frequency of collisions during autonomous operation:
  (18) $$CR=\frac{\mathrm{Number}\;\mathrm{of}\;\mathrm{Collisions}}{\mathrm{Total}\;\mathrm{Distance}\;\mathrm{Driven}}$$
  Expressed as collisions per kilometer, this metric evaluates the efficacy of collision avoidance systems integrated into autonomous driving algorithms.

These metrics collectively provide a robust framework for evaluating autonomous driving systems under varied driving conditions, thus facilitating a detailed analysis of their capability to navigate complex urban environments while adhering to traffic rules and maintaining high safety standards.

### 5.4. Baseline Setup

- Dreamer [39]: A reinforcement learning agent designed to tackle long-horizon tasks using latent imagination in learned world models. It distinguishes itself by employing deep learning to process high-dimensional sensory inputs and learn intricate behaviors.
- LatentSW-PPO [43]: Wang et al introduced a novel RL framework for autonomous driving that enhances safety and efficiency. This framework integrates a latent dynamic model that captures environmental dynamics from bird’s-eye view images, thereby improving learning efficiency and mitigating safety risks through synthetic data generation. Additionally, it incorporates state-wise safety constraints using a barrier function to ensure safety at every state during the learning process.
- Diffuser [35]: Janner et al proposed a novel approach to model-based reinforcement learning that integrates trajectory optimization into the modeling process, addressing the empirical shortcomings of traditional methods. They utilize a diffusion probabilistic model to plan by iteratively denoising trajectories, making sampling and planning nearly identical. In contrast to their proposed model, we further enhanced it with safety considerations.

Additionally, we propose several ablation versions of our method to evaluate the performance of each sub-module.

- Safe Autonomous Driving with Latent End-to-end Navigation (SAD-LEN ): This version excludes the latent diffusion component, relying solely on traditional latent state representation, the primary focus of which is on evaluating the impact of the latent state representation on navigation performance without the enhancements provided by diffusion processes.
- Autonomous Driving with End-to-end Navigation and Diffusion (AD-END): This version removes the safety guarantee mechanisms, focusing on the integration of end-to-end navigation with diffusion models. It aims to assess the contribution of safety constraints to overall performance and safety.

## 6. Results and Analysis

### 6.1. Evaluating Prediction Performance

**Figure 8:**
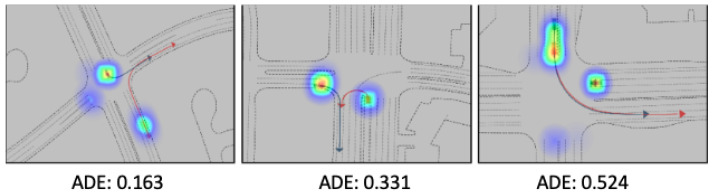
Visualization of the prediction for surrounding agents.

The accuracy of future scene generation is indeed crucial, as it directly impacts the safety and appropriateness of the actions taken. As shown in Figure 8, we can see that our model has better prediction and interoperability for surrounding agents. From the attention map, it indicates it can align with our logic.

### 6.2. Evaluating Safety and Efficiency During Exploratory

**Figure 9:**
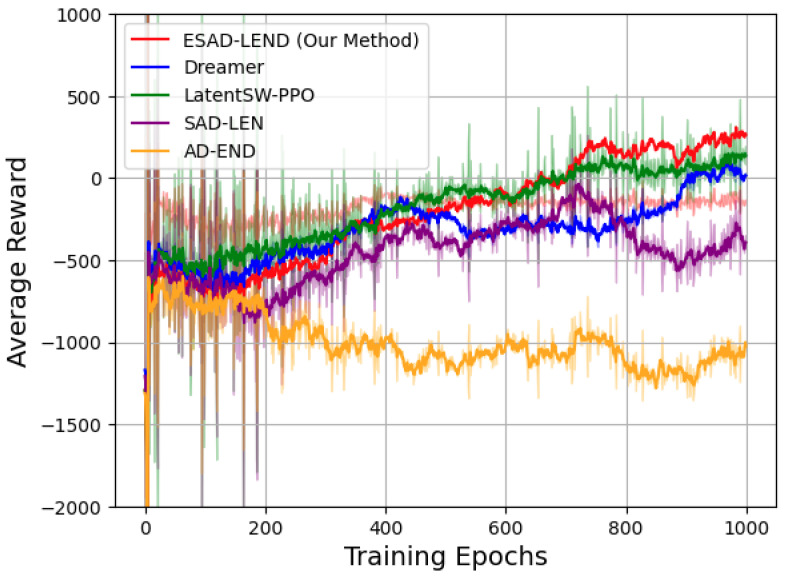
Comparative performance of various reinforcement learning methods over 1000 training epochs. The plot showcases the average reward trajectories for ESAD-LEND (our method), Dreamer, LatentSW-PPO, SAD-LEN, and AD-END.

We first evaluate the performance of the scenarios depicted in Figure 9. The primary evaluation criteria are derived from the metrics mentioned above, with a focus on the Average Displacement Error (ADE), which measures the average distance between the predicted trajectory and the actual trajectory of surrounding agents. Overall, the visualizations demonstrate that the model is capable of generating reasonable and accurate predictions across different scenarios.

In our comprehensive evaluation of autonomous driving systems, as shown in Table 1, ESAD-LEND demonstrated superior performance across multiple metrics in a simulated testing environment. ESAD-LEND achieved the highest DS (91.2%) and RC (98.3%), surpassing all comparative methods, including the baseline SAC, which had a DS of 78.2% and RC of 90.1%. Additionally, ESAD-LEND exhibited remarkable compliance and safety, recording the lowest IS (0.5%), indicative of fewer traffic violations and enhanced adherence to safety protocols. Its operational efficiency was also superior, with the lowest scores in CR and TTC, suggesting reduced incidences and smoother operational flow. Furthermore, its ability to handle unexpected obstructions was demonstrated by the lowest CO score (0.5%), highlighting its robustness and adaptability in dynamic environments. Diffuser also showed strong performance, with a DS of 87.6% and RC of 96.4%, and it recorded the lowest IS (0.3%), further emphasizing its reliability and effectiveness. These results establish ESAD-LEND and Diffuser as leading approaches in AI-driven transportation, providing safe, efficient, and compliant autonomous driving experiences.

### 6.3. Evaluate Generalization Ability

We conducted two additional experiments, one using a map from CARLA Table 2, and another in a real-world environment. (Table 3).

The comparative analysis of path planning algorithms in both simulated and real-world environments highlights the performance and reliability of the ESAD-LEND method. In the CARLA simulated environment, ESAD-LEND demonstrates superior performance across multiple metrics, achieving the highest DS of 95.3% with minimal variance, indicating consistent performance across trials. Additionally, it excels in RC at 99.2%, significantly ahead of other methods such as Dreamer and LatentSW-PPO, which score lower in both categories.

Furthermore, ESAD-LEND maintains the lowest IS and other critical safety metrics, including CO and Collision per Kilometer (CP), indicating fewer rule violations and safer driving behavior compared to the other models. These results underscore the robustness of ESAD-LEND in adhering to safety standards while effectively navigating complex environments.

### 6.4. Bridging the Gap between Simulation and Real-World

To bridge the gap between simulation and real-world experiments, our research considered a multi-faceted approach that addresses the sim-to-real transfer and quantifies the reality gap. Inspired by the work [44], we utilize the Pearson correlation coefficient (PCC) and the max normalized cross-correlation (MNCC) to evaluate the correlation between simulation results and real-world data, ensuring that our models perform consistently across both domains.

In real-world settings, the ESAD-LEND continues to demonstrate its efficacy by achieving the shortest path length and lowest maximum curvature among all tested algorithms, resulting in more efficient and smoother paths. Despite slightly longer training times compared to other methods, ESAD-LEND records the lowest failure rate, confirming its reliability and practical applicability in real-world scenarios where unpredictable variables can affect outcomes.

This comprehensive evaluation demonstrates that ESAD-LEND excels in controlled simulations and adapts effectively to real-world conditions, surpassing established algorithms in terms of both efficiency and safety.

### 6.5. Evaluate Robustness

**Figure 10:**
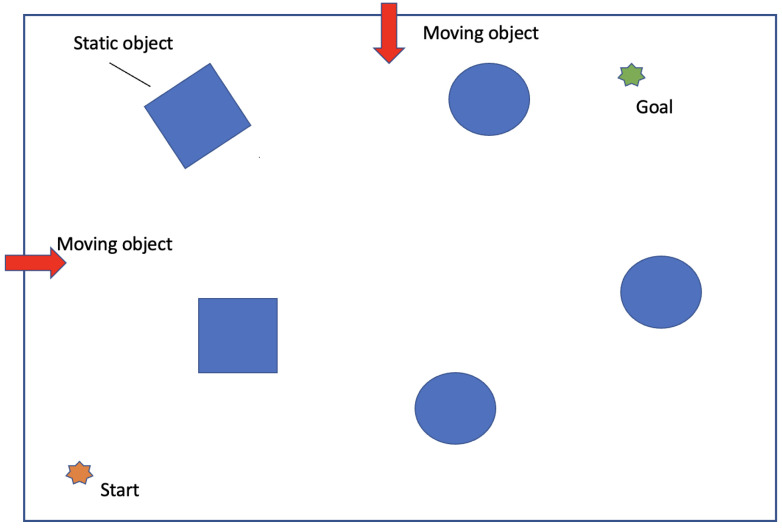
Scenario with static and moving objects to mimic real-world dynamics. Students simulate moving obstacles at speeds of 1 m/s, 2 m/s, and 3 m/s.

As shown in Figure 10, we further evaluate the robustness of our proposed framework against different types of obstacles. By adjusting the speed of moving obstacles, we simulate various levels of dynamic complexity and observe how well the model anticipates and reacts to changing trajectories and potential hazards. This test demonstrates the model’s obstacle avoidance skills and evaluates its potential real-world applicability and robustness under varying speeds and densities of pedestrian traffic. The speeds of the dynamic objects are set at 1 m/s, 2 m/s, and 3 m/s, with fixed start and goal points.

The Table 4 provides a detailed comparison of various path planning algorithms under dynamic conditions with varying speeds of moving obstacles. ESAD-LEND consistently outperforms other algorithms across all speed scenarios, demonstrating its superior ability to adapt and maintain high safety scores even with increasing obstacle speed. Notably, ESAD-LEND maintains a low failure rate, with only a slight increase as the obstacle speed escalates from 1 m/s to 3 m/s. This robust performance underscores its effective handling of dynamic challenges in real-world environments.

Dreamer demonstrates reasonable performance at lower speeds but exhibits a significant drop in safety scores and an increase in failure rates as the speed increases, indicating limited adaptability to faster-moving obstacles. Similarly, LatentSW-PPO and SAD-LEN perform adequately at lower speeds but struggle to maintain efficiency and safety at higher speeds.

AD-END and SAC, designed for robustness, show the highest failure rates and longest completion times, particularly at higher speeds, suggesting areas for improvement in their algorithms to better handle dynamic and unpredictable environments.

Overall, the analysis highlights that while most algorithms manage slower-moving obstacles adequately, the primary challenge lies in adapting to higher speeds, where reaction times and predictive capabilities are crucial. ESAD-LEND’s superior performance underscores its effectiveness in minimizing risks and optimizing path planning across varied dynamic conditions.

## 7. Conclusions

In this study, we propose a novel end-to-end algorithm for autonomous driving employing safe reinforcement learning. We develop a latent imagination model to forecast future trajectories, enabling the agent to explore within an imagined horizon initially. This method mitigates irreversible damage during training. Additionally, we introduce a VaR-based soft actor-critic to address the constrained optimization problem. Our model-based reinforcement learning approach demonstrates robustness and achieves a balanced trade-off between exploration and exploitation. In experiments, we validate the Carla simulator and real-world environments.

In future research, we plan to utilize other simulators such as Highway [45] to expand testing scenarios. Additionally, we aim to explore multi-agent settings where agents collaborate. We will also consider more complex network structures such as the graph attention algorithm [46,47].

## Figures and Tables

**Table 1:** Enhanced driving performance and infraction analysis in the testing environment with variance indicators.

Metric	ESAD-LEND	Dreamer	LatentSW-PPO	SAD-LEN	AD-END	SAC	Diffuser
DS (%)	91.2 ± 1.5	91.7 ± 2.1	86.5 ± 2.4	83.3 ± 2.7	80.4 ± 2.9	78.2 ± 3.1	87.6 ± 2.3
RC (%)	98.3 ± 0.5	97.2 ± 0.7	95.8 ± 0.9	94.1 ± 1.1	92.0 ± 1.3	90.1 ± 1.6	96.4 ± 0.8
IS (%)	0.5 ± 0.1	0.7 ± 0.2	0.9 ± 0.2	1.1 ± 0.3	0.4 ± 0.3	1.6 ± 0.4	0.9 ± 0.3
CO (%)	0.5 ± 0.1	0.8 ± 0.2	1.0 ± 0.2	1.3 ± 0.3	1.6 ± 0.4	1.9 ± 0.5	0.7 ± 0.2
CR (%)	0.4 ± 0.1	0.6 ± 0.2	0.8 ± 0.2	1.0 ± 0.3	1.3 ± 0.4	1.5 ± 0.5	0.6 ± 0.2
TTC (%)	0.4 ± 0.1	0.6 ± 0.2	0.8 ± 0.2	1.0 ± 0.3	1.3 ± 0.4	1.5 ± 0.5	0.6 ± 0.2

**Table 2:** Enhanced driving performance and infraction analysis in the testing environment in CARLA with variance indicators.

Metric	ESAD-LEND	Dreamer	LatentSW-PPO	SAD-LEN	AD-END	SAC	Diffuser
DS (%)	95.3 ± 1.2	87.4 ± 2.6	84.1 ± 3.0	80.5 ± 3.5	78.9 ± 3.8	76.8 ± 4.1	85.6 ± 5.5
RC (%)	99.2 ± 0.3	96.5 ± 1.1	94.3 ± 1.4	92.1 ± 1.7	89.8 ± 2.0	87.6 ± 2.4	96.4 ± 0.9
IS (%)	0.4 ± 0.05	0.7 ± 0.1	1.0 ± 0.15	1.2 ± 0.18	1.5 ± 0.22	1.8 ± 0.25	0.3 ± 0.1
CO (%)	0.2 ± 0.03	0.6 ± 0.09	0.9 ± 0.13	1.2 ± 0.16	1.5 ± 0.20	1.8 ± 0.24	0.5 ± 0.2
CR (%)	0.2 ± 0.04	0.5 ± 0.08	0.7 ± 0.11	0.9 ± 0.13	1.2 ± 0.16	1.4 ± 0.19	0.4 ± 0.1
TTC (%)	0.3 ± 0.05	0.6 ± 0.09	0.9 ± 0.13	1.1 ± 0.16	1.4 ± 0.19	1.6 ± 0.22	0.4 ± 0.2

**Table 3:** Comparison of path planning algorithms in a real-world environment.

Planning Algorithm	Length of Path (m)	Maximum Curvature	Training Time (min)	Failure Rate (%)
ESAD-LEND	44.2	0.48	89	3
Dreamer	46.7	0.60	160	7
LatentSW-PPO	45.5	0.43	155	4
SAD-LEN	47.9	0.66	170	9
AD-END	46.3	0.58	165	11
SAC	48.1	0.72	160	14
Diffuser	45.0	0.50	140	5

**Table 4:** Performance comparison of path planning algorithms under varying speed scenarios.

Speed	1 m/s			2 m/s			3 m/s		
**Algorithm**	**Fail Rate** **(%)**	**Avg. Time** **(s)**	**Safety** **Score**	**Fail Rate** **(%)**	**Avg. Time** **(s)**	**Safety** **Score**	**Fail Rate** **(%)**	**Avg. Time** **(s)**	**Safety** **Score**
ESAD-LEND	1	120	95	3	130	93	5	140	90
Dreamer	5	140	90	7	150	88	10	170	85
LatentSW-PPO	3	130	93	5	140	90	8	160	87
SAD-LEN	4	135	92	6	145	89	9	165	86
AD-END	7	150	88	10	160	85	14	180	82
SAC	6	145	89	9	155	87	13	175	84
Diffuser	2	125	94	4	135	91	6	150	88

## Data Availability

Data are contained within the article.
